# A Ductal-Cell-Related Risk Model Integrating Single-Cell and Bulk Sequencing Data Predicts the Prognosis of Patients With Pancreatic Adenocarcinoma

**DOI:** 10.3389/fgene.2021.763636

**Published:** 2022-01-03

**Authors:** Xitao Wang, Xiaolin Dou, Xinxin Ren, Zhuoxian Rong, Lunquan Sun, Yuezhen Deng, Pan Chen, Zhi Li

**Affiliations:** ^1^ Xiangya Cancer Center, Xiangya Hospital, Central South University, Changsha, China; ^2^ Key Laboratory of Molecular Radiation Oncology Hunan Province, Changsha, China; ^3^ National Clinical Research Center for Geriatric Disorders, Xiangya Hospital, Central South University, Changsha, China; ^4^ Department of Pancreatic Surgery, Xiangya Hospital, Central South University, Changsha, China; ^5^ Hunan Cancer Hospital and the Affiliated Cancer Hospital of Xiangya School of Medicine, Central South University, Changsha, China

**Keywords:** single-cell sequencing, bulk sequencing, prognosis, pancreatic adenocarcinoma, risk model

## Abstract

Pancreatic ductal adenocarcinoma (PDAC) is a highly heterogeneous malignancy. Single-cell sequencing (scRNA-seq) technology enables quantitative gene expression measurements that underlie the phenotypic diversity of cells within a tumor. By integrating PDAC scRNA-seq and bulk sequencing data, we aim to extract relevant biological insights into the ductal cell features that lead to different prognoses. Firstly, differentially expressed genes (DEGs) of ductal cells between normal and tumor tissues were identified through scRNA-seq data analysis. The effect of DEGs on PDAC survival was then assessed in the bulk sequencing data. Based on these DEGs (*LY6D, EPS8, DDIT4, TNFSF10, RBP4, NPY1R, MYADM, SLC12A2, SPCS3, NBPF15*) affecting PDAC survival, a risk score model was developed to classify patients into high-risk and low-risk groups. The results showed that the overall survival was significantly longer in the low-risk group (*p* < 0.05). The model also revealed reliable predictive power in different subgroups of patients. The high-risk group had a higher tumor mutational burden (TMB) (*p* < 0.05), with significantly higher mutation frequencies in *KRAS* and *ADAMTS12* (*p* < 0.05). Meanwhile, the high-risk group had a higher tumor stemness score (*p* < 0.05). However, there was no significant difference in the immune cell infiltration scores between the two groups. Lastly, drug candidates targeting risk model genes were identified, and seven compounds might act against PDAC through different mechanisms. In conclusion, we have developed a validated survival assessment model, which acted as an independent risk factor for PDAC.

## Introduction

Pancreatic cancer is a highly aggressive malignant tumor of the digestive system, 95% of which are pancreatic ductal adenocarcinoma (PDAC). In recent years, its incidence and mortality have increased by an average of 0.3% per year with lifestyle changes and factors such as increased life expectancy and an aging population (Santucci et al., 2020). Early diagnosis of pancreatic cancer is challenging due to the lack of specific symptoms and biological markers. Surgery remains the only possible cure for pancreatic cancer, but approximately 80–85% of patients present with either unresectable or metastatic disease at the time of diagnosis (Mizrahi et al., 2020). Molecular events in tumors usually precede the presentation of clinical features. Thus, effective molecular markers can more accurately predict patient prognosis and suggest individualized treatment plans.

Recent technological advances have enabled researchers to use a variety of sequencing methods to identify somatic variants, methylation changes, and other genomic alterations in tumors (Malone et al., 2020). However, traditional bulk sequencing technologies target all cells in a sample and can only reflect the average level of variation of the tumor. The advancement of single-cell sequencing (scRNA-seq) technology has provided researchers with a view into cancer at unprecedented molecular resolution. With the increasing emphasis on intra-tumor heterogeneity, scRNA-seq has emerged as a powerful tool to reveal the unique genetic information of each cell and discover new cell types (Suvà and Tirosh, 2019). The application of this technology helps to uncover tumor characteristics previously hidden in cell population heterogeneity, which might provide potential prognostic biomarkers for better clinical decisions in individualized treatment (Papalexi and Satija, 2018). But scRNA-seq studies have limited clinical samples that cannot be correlated with clinical data from a large number of patients (e.g., prognostic information). In this case, considering a large amount of complete clinical information available in bulk sequencing cohorts, an appropriate combination of scRNA-seq and bulk sequencing results would optimize the utilization of these data.

In this study, we used scRNA-seq data to screen potential prognostic genes for PDAC. By comparing the differences in ductal cell gene expression between normal and tumor tissues, we identified 10 genes affecting the overall survival (OS) of PDAC. A prognostic risk model based on these ductal cell features was then developed and further validated using external bulk sequencing data. Multiple bioinformatics methods were applied to analyze the molecular characteristics of patients classified by this prognostic model, and drug candidates targeting these prognostic-related genes were also identified.

## Methods

### Data Source

Our study applied a comprehensive analysis of data publicly available online. The scRNA-seq dataset (PRJCA001063) containing 24 PDAC tumor samples and 11 control pancreases without any treatment was obtained from the Zenodo database (www.zenodo.org) (Peng et al., 2019). Bulk sequencing data in the training and validation sets were obtained from The Cancer Genome Atlas (TCGA) (TCGA- PAAD) (https://portal.gdc.cancer, updated until 07-20-2019), the Gene Expression Omnibus (GEO) (GSE71729, GSE21501) (https://www.ncbi.nlm.nih.gov/geo/query), the European Molecular Biology Laboratory (EMBL-EBI) (E-MTAB-6134) (https://www.ebi.ac.uk/arrayexpress/experiments/E-MTAB-6134/) and the International Cancer Genome Consortium Data Portal (ICGC) Canada pancreatic cancer project (PACA-CA) (ICGC-CA, https://dcc.icgc.org/releases/current/Projects/PACA-CA). Mutation data in TCGA cases were downloaded from the Broad Institute TCGA Genome Data Analysis Center (http://gdac.broadinstitute.org). Transcriptomic and clinical information for each dataset was simultaneously downloaded from the respective websites when available. Only PDAC was included in the subsequent analysis, while other histological subtypes, such as Acinar Cell Carcinoma, adenosquamous carcinoma, mucinous cystadenocarcinoma, mixed ductal endocrine carcinoma were excluded.

### Processing of scRNA-seq Data

We used the “Seurat” package deployed in R for quality control and downstream analysis of scRNA-seq data (Hao et al., 2021). The regularizing negative binomial regression was used to eliminate batch effects. Low quality cells (<200 genes/cell, <3 cells/gene and >10% mitochondrial genes) were excluded. Afterward, we calculated the standardized variance of each gene across cells to generate highly variable genes and used them for principal component analysis (PCA). “ElbowPlot” analysis and heat map visualization in “Seurat” were used to identify significant principal components (PCs). Based on PC1 to PC13, graph-based clustering was applied (res = 0.8) to identify different cell groups. Then, non-linear dimensionality reduction was performed using the “tSNE” method. Different cell clusters were identified and annotated with the “singleR” package, the CellMarker database, and previously published scRNA-seq analysis (Peng et al., 2019), (Aran et al., 2019; Zhang et al., 2019; Schlesinger et al., 2020). To identify differentially expressed genes (DEGs) between ductal cells in normal and tumor tissues, we used the “FindMarkers” function in the “Seurat” package. DEGs were filtered by |log2 (fold change) | > 0.5 and *p* < 0.05.

### Construction and Validation of a Duct-Cell-Related Risk Model

Firstly, a univariate Cox regression analysis was performed on the TCGA-PDAC cohort to determine the association between the aforementioned duct-cell-related DEGs and OS. Then, significant DEGs related to OS (*p* < 0.05) were included in the least absolute shrinkage and selection operator (LASSO) regression analysis to reduce multicollinearity and make the model simpler and more effective. Finally, a multivariate Cox regression analysis was conducted on the screened genes to assess the impact of each gene as an independent prognostic factor on patient survival. The risk score formula was constructed as described in the previous study (Ma et al., 2018). We used the median risk score as a cut-off value to categorize the patients in the TCGA training set into high-risk and low-risk groups. Patients in the GEO validation group were also divided into two groups by the same method. Survival differences between the two groups were assessed by the Kaplan-Meier method and log-rank test. Furthermore, we plotted the time-dependent receiver operating characteristic (ROC) curves with 1, 3, and 5 years as the defined points and calculated the corresponding area under the ROC curve (AUC) to assess and compare the predictive power of the risk model with other recently published PADC risk models. The values of AUC range from 0.5 to 1, with 1 indicating full discriminant and 0.5 indicating no discriminant.

### Correlations Between the Risk Score and Clinical Features

Several clinical characteristics of PDAC patients might affect prognosis. To investigate whether the risk score was independent of relevant clinical factors (e.g., age, alcohol history, gender, chronic pancreatitis history, TNM stage, histologic grade, etc.), we performed univariate and multivariate Cox regression analyses. The significance level was set at *p* < 0.05.

### Gene Set Enrichment Analysis

To clarify the differences in signaling pathways and molecular mechanisms associated with gene expression profiles between high-risk and low-risk groups, we analyzed the gene expression data using GSEA analysis. Enriched gene sets with *p* < 0.05 and FDR <0.25 were considered statistically significant.

### Tumor Mutation Burden and Somatic Mutation Analysis

TMB was defined as the total number of mutations (changes) found in the DNA of cancer cells (Chan et al., 2019). The TMB of patients with mutation data in the TCGA cohort was calculated using the “tmb” function in the “MAFTOOLS” package (Mayakonda et al., 2018). Somatic mutation comparison was also completed with the same package for patients in the high- and low-risk groups. *p* < 0.05 was set as the level of significance.

### Assessment of Tumor Immune Microenvironment and Immunotherapeutic Response

The immune score for each patient in the TCGA cohort was calculated using the ESTIMATE algorithm. The standardized expression profile was then uploaded to the TIMER website (http://timer.cistrome.org/) to obtain an assessment of immune cell infiltration (including B cells, macrophage, myeloid dendritic cells, neutrophil, CD4^+^ T cells, and CD8^+^ T cells) (Li et al., 2020). We also applied the Tumor Immune Dysfunction and Exclusion (TIDE) algorithm to predict the patient’s response to immunotherapy (Fu et al., 2020). At a significance level of *p* < 0.05, we compared immune scores, degree of immune cell infiltration, and response to immunotherapy between the high-risk and low-risk groups.

### Estimation of Tumor Stemness and Drug Candidate Prediction

Stemness features were extracted from transcriptomic data of TCGA PDAC patients by using an innovative one-class logistic regression (OCLR) machine-learning algorithm (Malta et al., 2018). The transcriptome-based stemness index (mRNAsi) was then mapped to a range of 0–1 by linear transformation (subtracting the minimum and dividing by the maximum). To predict which compounds were likely to be effective against PDAC, we used Broad institute’s Connectivity Map (CMap) to screen drug candidates based on key genes of the risk model (Subramanian et al., 2017).

## Results

### DEGs Between Normal and Malignant Pancreatic Ductal Cells

**FIGURE 1:**
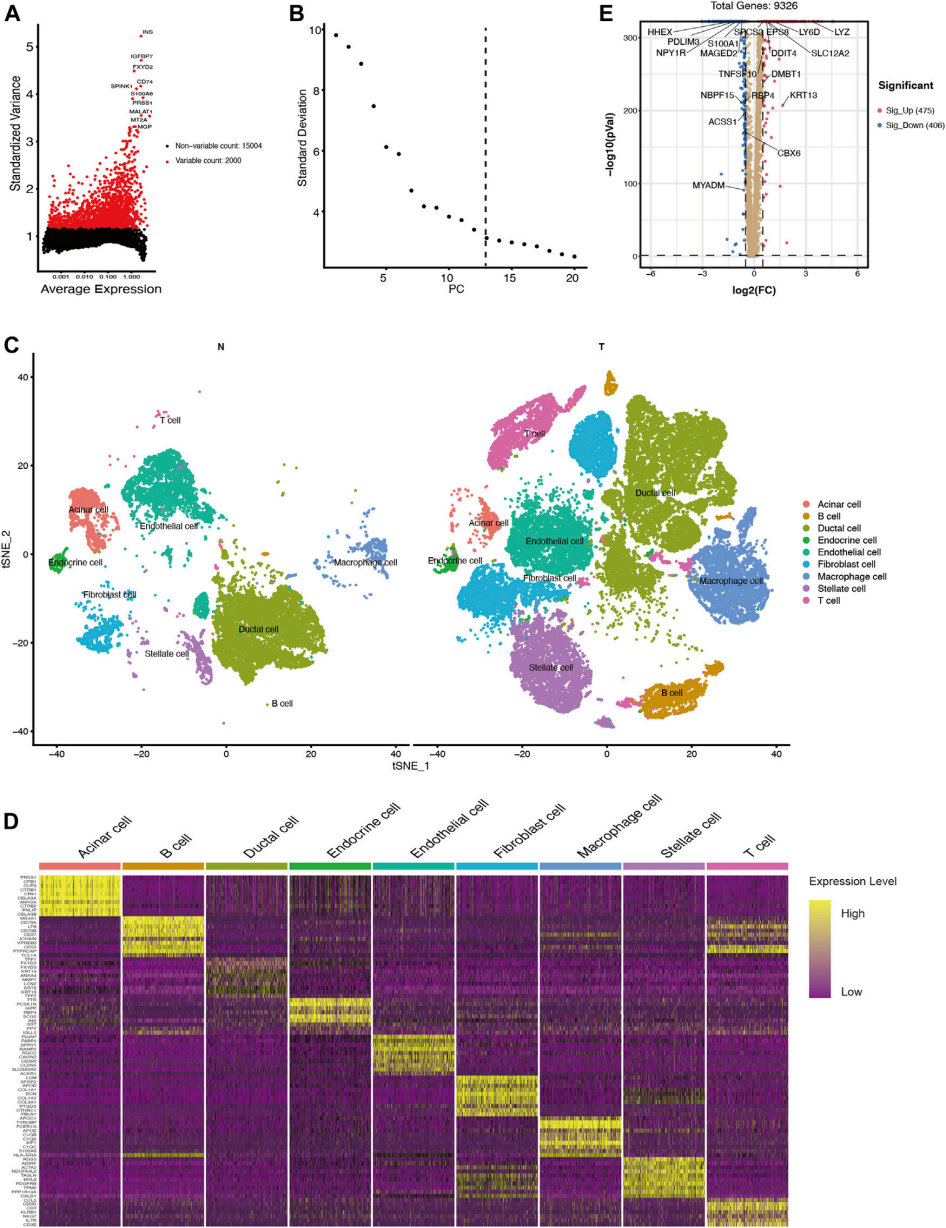
scRNA-seq identifies 9 cell types in the PDAC tissues and 881 differentially expressed genes between ductal cells in normal and tumor tissues. **(A)** The top 2000 variable genes with large, standardized variances for subsequent analysis. **(B)** The elbow plot shows 13 PCs appropriate for further cell cluster classification **(C)** tSNE algorithm classified cell clusters based on transcriptome data. **(D)** Heatmap of top 10 marker genes for each cell cluster. **(E)** Differentially expressed genes between ductal cells in normal and tumor tissues. T, tumor; N, normal.

To investigate changes of ductal cells in PDAC, we reanalyzed the scRNA-seq dataset and annotated the cell types according to their transcriptomic characteristics. After performing cell quality control (as described in the methods section), we performed principal component analysis using the top 2000 variable genes (Figure 1A) and identified 13 principal components for downstream analysis via elbow plots (Figure 1B). Then these cells were divided into 33 clusters (Supplementary Figure S1) and based on marker gene expression (Supplementary Figure S1, Supplementary Tables S1, S2) these clusters were classified into acinar cells, ductal cells, endothelial cells, endocrine cells, fibroblasts, stellate cells, T cells, B cells, and macrophages (Figures 1C,D). By comparing the transcriptional data of normal and malignant pancreatic ductal cells, we identified 881 differentially expressed genes (log2FC >0.5, *p* < 0.05), of which 475 were upregulated and 406 were downregulated (Figure 1E, SupplementaryTable S3).

### Construction of Risk Model

**FIGURE 2:**
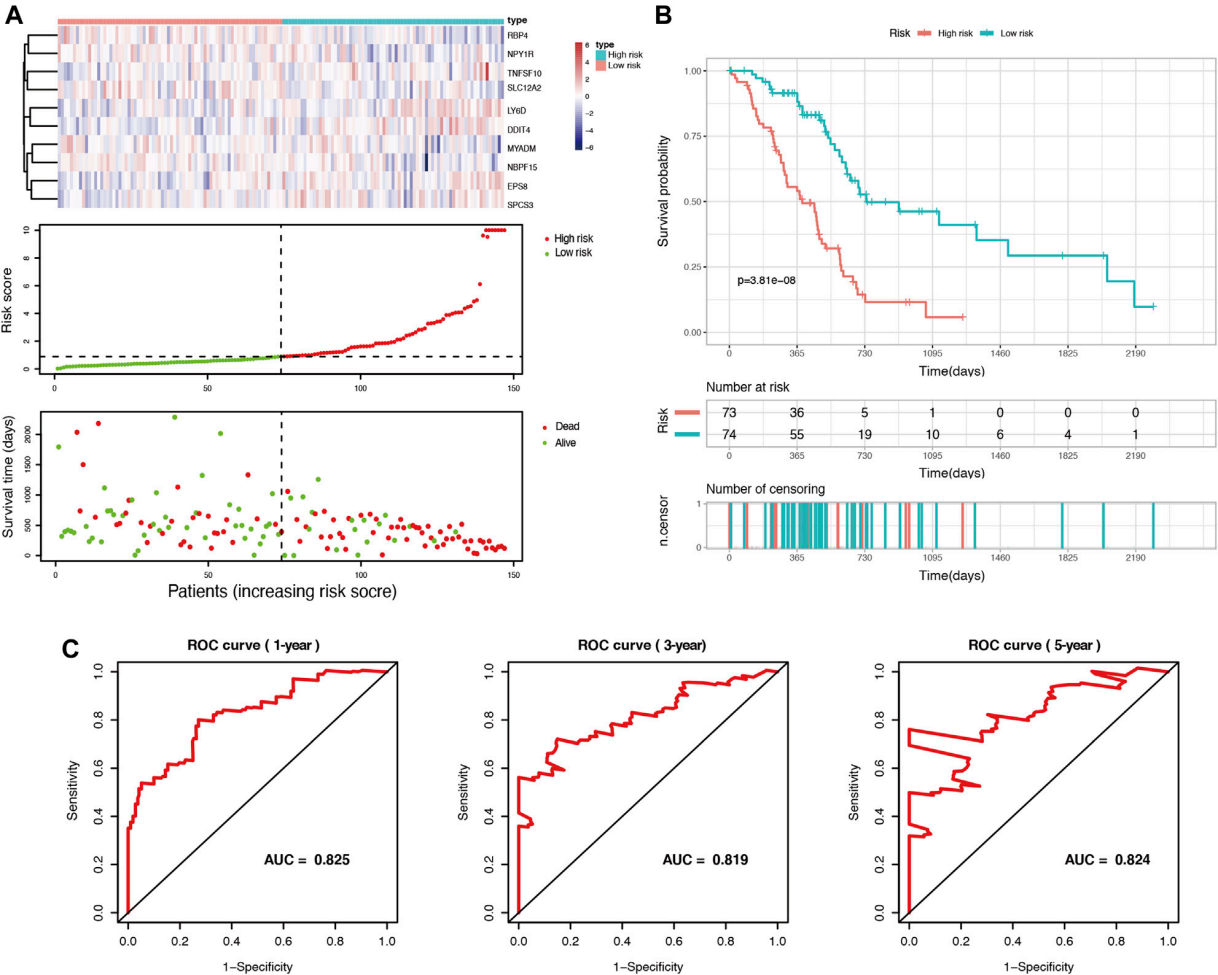
Identification of prognosis-related genes and construction of a duct-cell-related risk model. **(A)** Risk score analysis in the TCGA training set. Upper panel: heatmap of gene expressions in the PDAC samples. Middle panel: risk score curve based on the ductal-cell-related genes signature. Bottom panel: patient survival status and time distributed by risk score. **(B)** Kaplan-Meier survival curve of the risk score for patient OS in the TCGA cohort. **(C)** The prognostic performance of the risk model is demonstrated by the time-dependent ROC curve for predicting the 1-, 3-, and 5-years OS rates in the TCGA training set.

Among these DEGs, we used univariate Cox regression to initially screen out 98 genes associated with PDAC prognosis (*p* < 0.05) in the TCGA cohort. Then, these genes were included in LASSO regression analysis to further exclude confounding factors (Supplementary Figures S3A,B). Next, we performed a multivariate Cox regression analysis and select 10 genes to construct a prognostic prediction model (Supplementary Figure S3C, Supplementary Table S4). According to this model, we calculated the risk scores of 147 patients in the TCGA PDAC group. Using the median risk score as the cutoff point, all patients in the set were divided into a high-risk group and a low-risk group (Figure 2A). The median OS survival was 313 and 505 days for the high-risk and low-risk groups, respectively. Kaplan-Meier analysis showed that high-risk PDAC patients had significantly lower OS than low-risk PDAC patients (*p* < 0.001, Figure 2B). Then, the AUCs were calculated to assess the OS prediction efficiency of the risk model. It had AUCs of 0.825, 0.819, and 0.824 at 1, 3, and 5 years, indicating that this model had favorable predictive power (Figure 2C).

**FIGURE 3:**
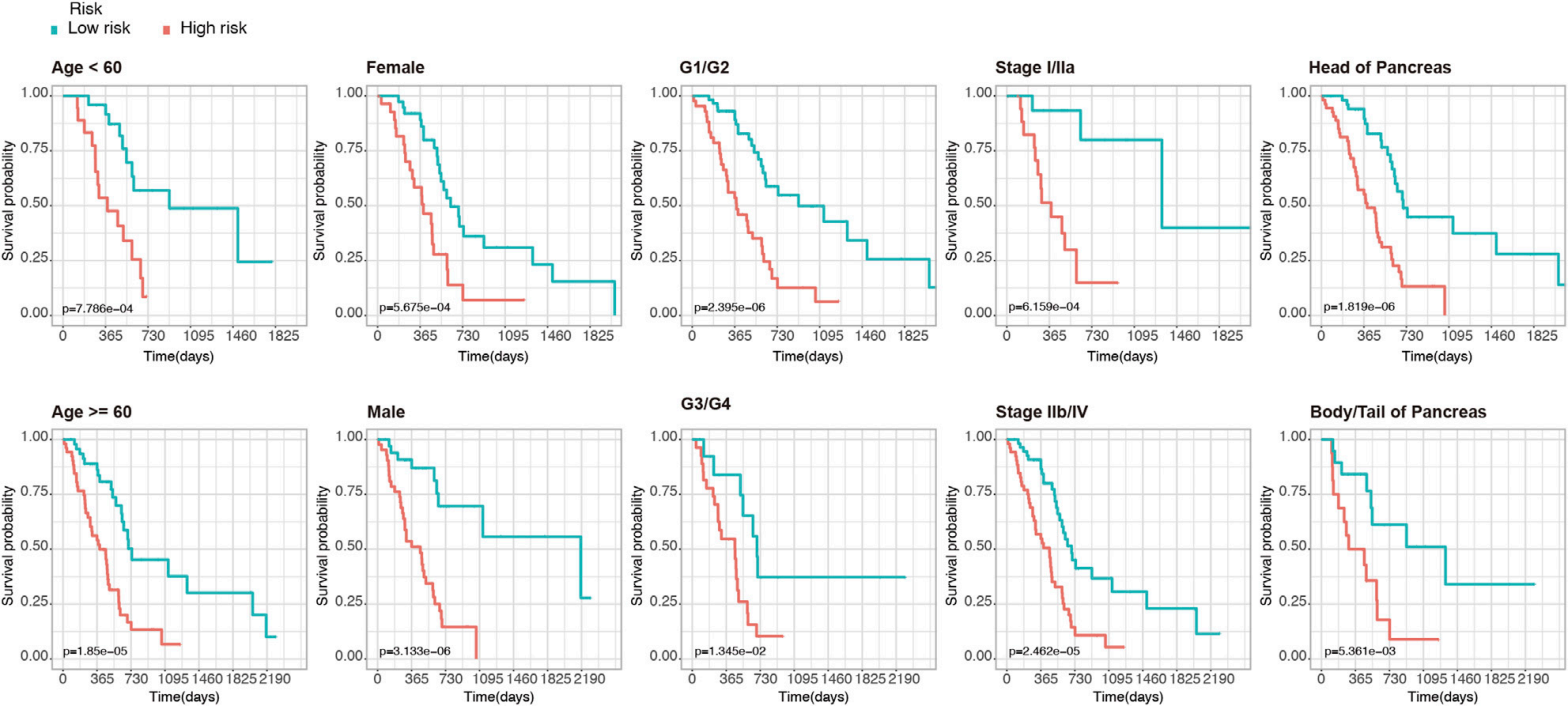
Kaplan-Meier analysis of OS for PDAC patients in the TCGA set. Patients were classified according to age (age <60 and age ≥60), sex (male and female), tumor histologic grade (G1/G2 and G3/G4), TNM stage (stage I/IIa and stage IIb/IV), and anatomical location of the lesion (head of pancreas and body/tail of Pancreas).

Furthermore, to clarify the reliability of the risk model, we divided the PDAC patients in the TCGA dataset into subgroups with different clinical characteristics (including age, gender, histologic grade, AJCC stage, and tumor location). Kaplan-Meier analysis showed that even among the different subgroups, patients in the high-risk group had significantly lower OS than those in the low-risk group (*p* < 0.05, Figure 3), which further demonstrated the superiority of this model.

### Validation of the Risk Model in External Cohorts

**FIGURE 4:**
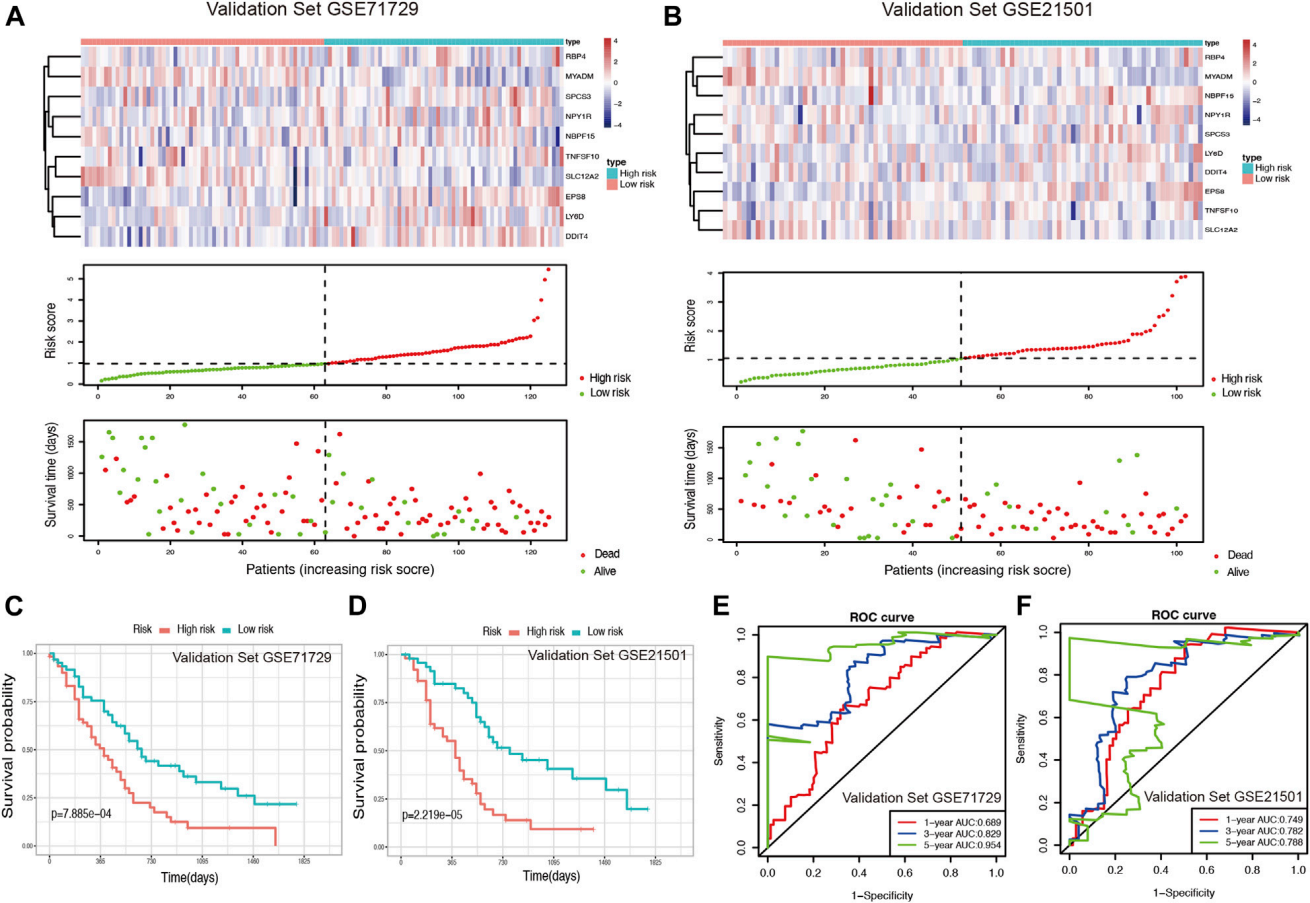
Validation of the risk model for PDAC survival using GEO datasets. **(A,B)** Risk score analysis in the validation sets GSE71729 and GSE21501. **(C,D)** Kaplan-Meier survival curve of the risk score for patient OS in the validation sets GSE71729 and GSE21501. **(E,F)** The time-dependent ROC curve for predicting the 1-, 3-, and 5-years OS rates in the validation sets GSE71729 and GSE21501.

Using two GEO datasets (GSE71729 and GSE21501), E-MTAB-6134 and ICGC-CA, we validated the reliability and stability of the ductal cell-related risk model. Risk scores were calculated in both cohorts and PDAC patients were then divided into high-risk and low-risk groups (Figures 4A,B, Supplementary Figures S5A,B). Consistent with the results of the training set, the OS of high-risk patients in both validation sets was significantly shorter than that of low-risk patients (*p* < 0.001, Figures 4C,D, Supplementary Figures S5C,D). In GSE71729, the median OS of high-risk patients and low-risk patients were 300 and 540 days, while they were 330 and 540 days in GSE21501, 483 and 757 days in E-MTAB-6134, and 445 and 590 days in ICGC-CA, respectively. The results of the AUC analysis also showed that the model had favorable predictive power in the validation sets. In GSE71729, the AUCs were 0.689, 0.829, and 0.954 for 1, 3, and 5 years, while they were 0.749, 0.782, and 0.788 for 1, 3, and 5 years in GSE21501, 0.713, 0.665, and 0.686 for 1, 3, and 5 years in E-MTAB-6134, and 0.675, 0.705, and 0.749 for 1, 3, and 5 years in ICGC-CA, respectively (Figures 4E,F, Supplementary Figure S6).

To further validate the predictive power of the ductal cell risk model, we compared it with three recently published pancreatic cancer risk scoring models, including the Deng model (Deng et al., 2021), the Qiu model (Qiu et al., 2020), and the Wu model (Wu et al., 2019). Notably, these three models were all built on bulk sequencing data. Few pancreatic cancer risk models based on single cell data have been reported. We compared the predictive power of each model by the area under the ROC curve (AUC). In general, the ductal cell model based on single cell RNA sequencing possessed better predictive power in all four validation datasets, especially in terms of 5-years overall survival prediction (see Supplementary Figure S6).

### Correlations Between the Risk Score and Clinical Features

**FIGURE 5:**
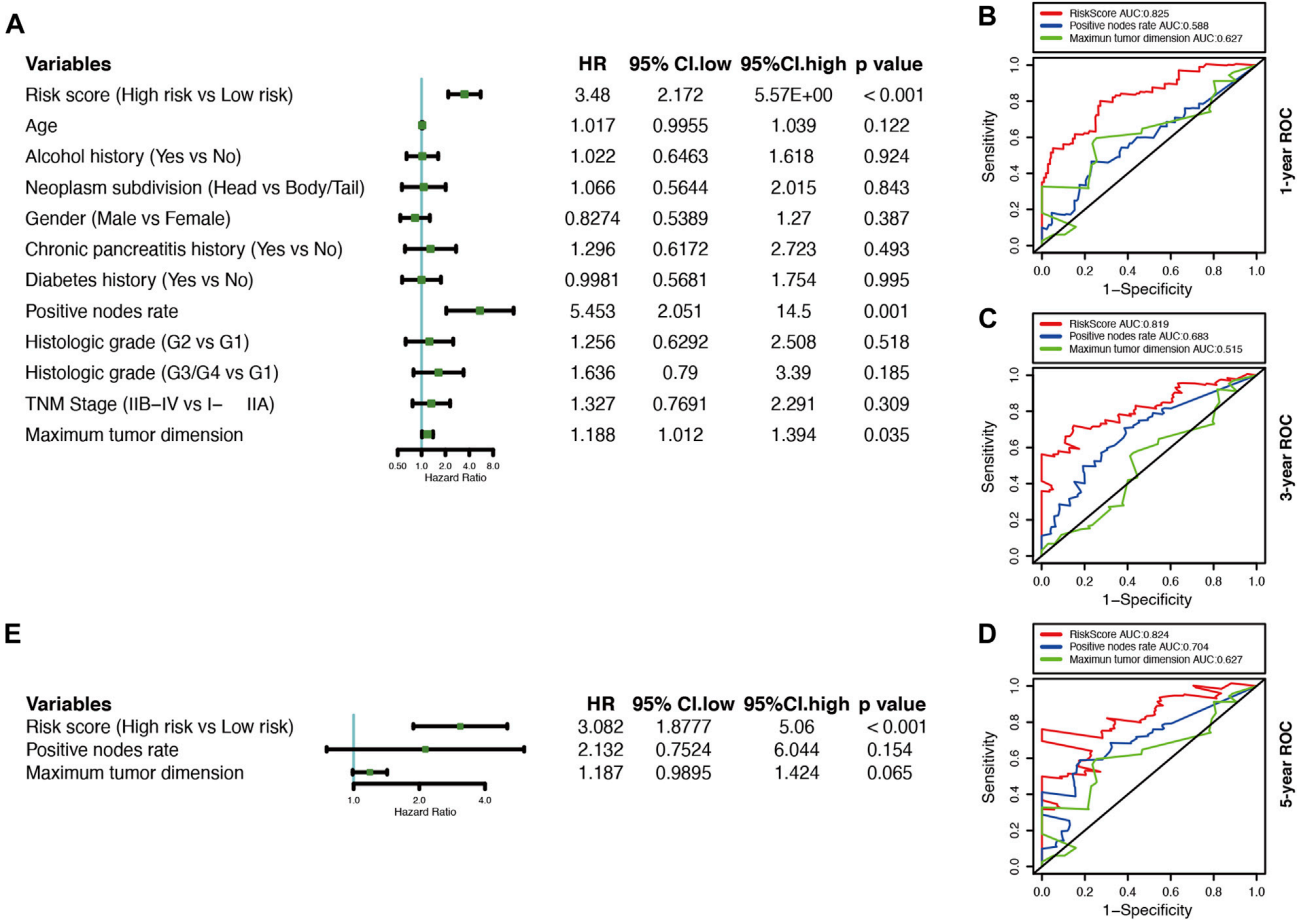
Correlations between the risk model and clinical characteristics with OS based on the TCGA cohort. **(A)** Univariate Cox analysis of clinical characteristics and the risk score. **(B–D)** Comparison of time-dependent ROC curves for predicting the 1-, 3-, and 5-years OS rates among the maximum tumor dimension, positive nodes rate, and risk score.

A univariate Cox regression analysis was performed to explore the relationship between patient clinical features and PDAC prognosis in the TCGA cohort. The results showed that only the positive nodes rate (HR, 5.453; 95% CI, 2.051 to 14.500; *p* = 0.001), as well as the maximum tumor dimension (HR, 1.188; 95% CI, 1.102 to1.394; *p* = 0.035), were significantly associated with the prognosis of TCGA-PDAC patients (Figure 5A). Then through ROC analysis, we calculated the AUCs of risk score, positive nodes rate, and maximum tumor dimension at 1, 3, and 5 years (Figures 5B–D). The results demonstrated that the predictive efficacy of the risk score was better than that of positive nodes rate and maximum tumor dimension. The risk score also acted as an independent risk factor for the PDAC prognosis in a multivariate Cox regression analysis (*p* < 0.001) (Figure 5E).

### Gene Set Enrichment Analysis

**FIGURE 6:**
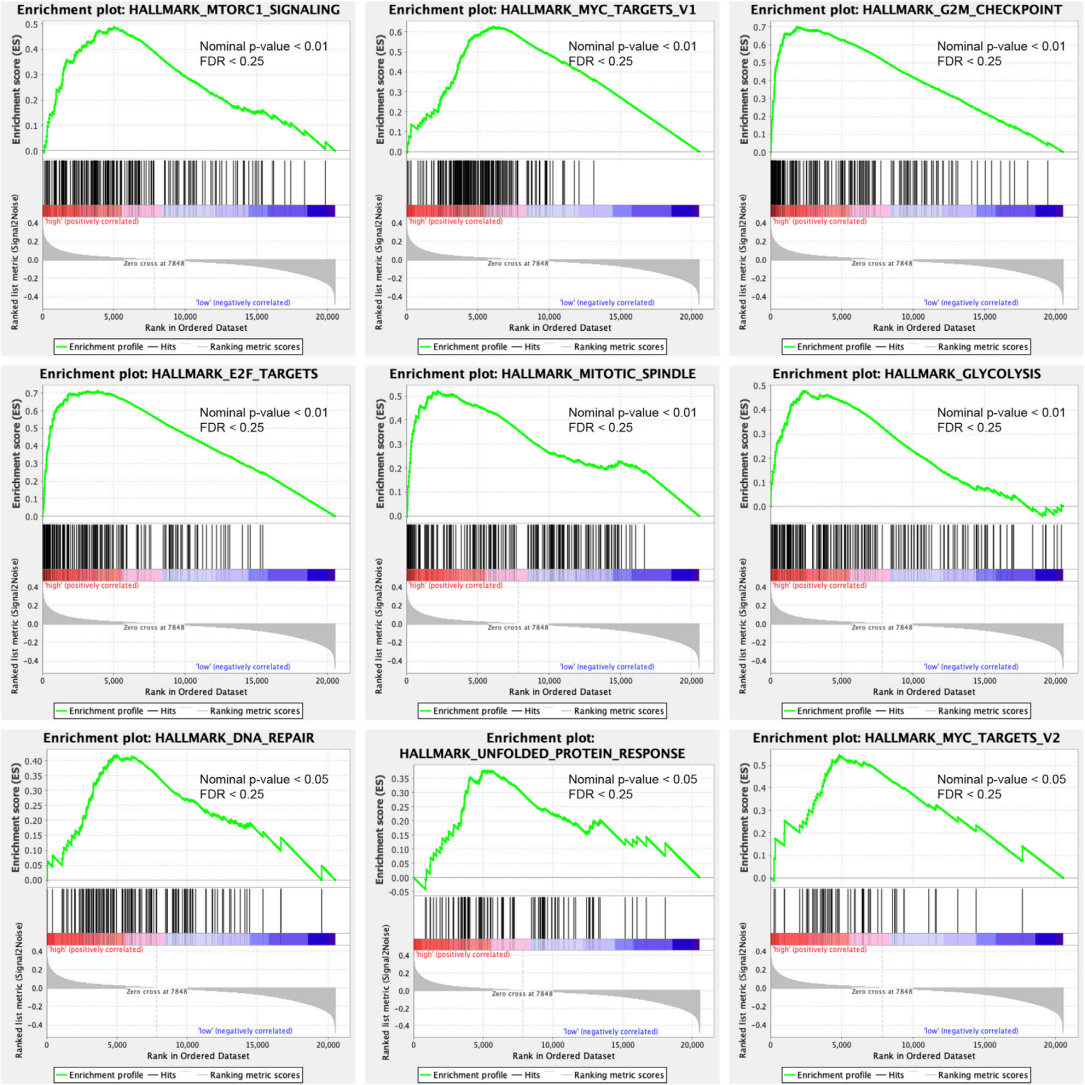
Gene set enrichment analysis indicated significant enrichment of hallmark cancer-related pathways in the high-risk group based on the TCGA dataset. mTORC1, mammalian target of rapamycin complex 1.

GSEA analysis was conducted to annotate the function of DEGs between high-risk and low-risk patient groups. 9 cancer-related gene sets were demonstrated to be significantly enriched in the high-risk patient group (Nominal *p*-value < 0.05, FDR <0.25), including mTORC1 signaling, MYC targets v1, MYC targets v2, G2M checkpoint, E2F targets, mitotic spindle, glycolysis, DNA repair, and unfolded protein response (Figure 6). The gene sets were found to be intimately involved in tumorigenesis, DNA repair, genome stability, and tumor nutrition and metabolism (Stine et al., 2015; Kent and Leone, 2019; Murugan, 2019; Yang et al., 2020). The result might provide clues to the potential mechanisms affecting the prognosis of PDAC patients.

### Analysis of Somatic Mutations

**FIGURE 7:**
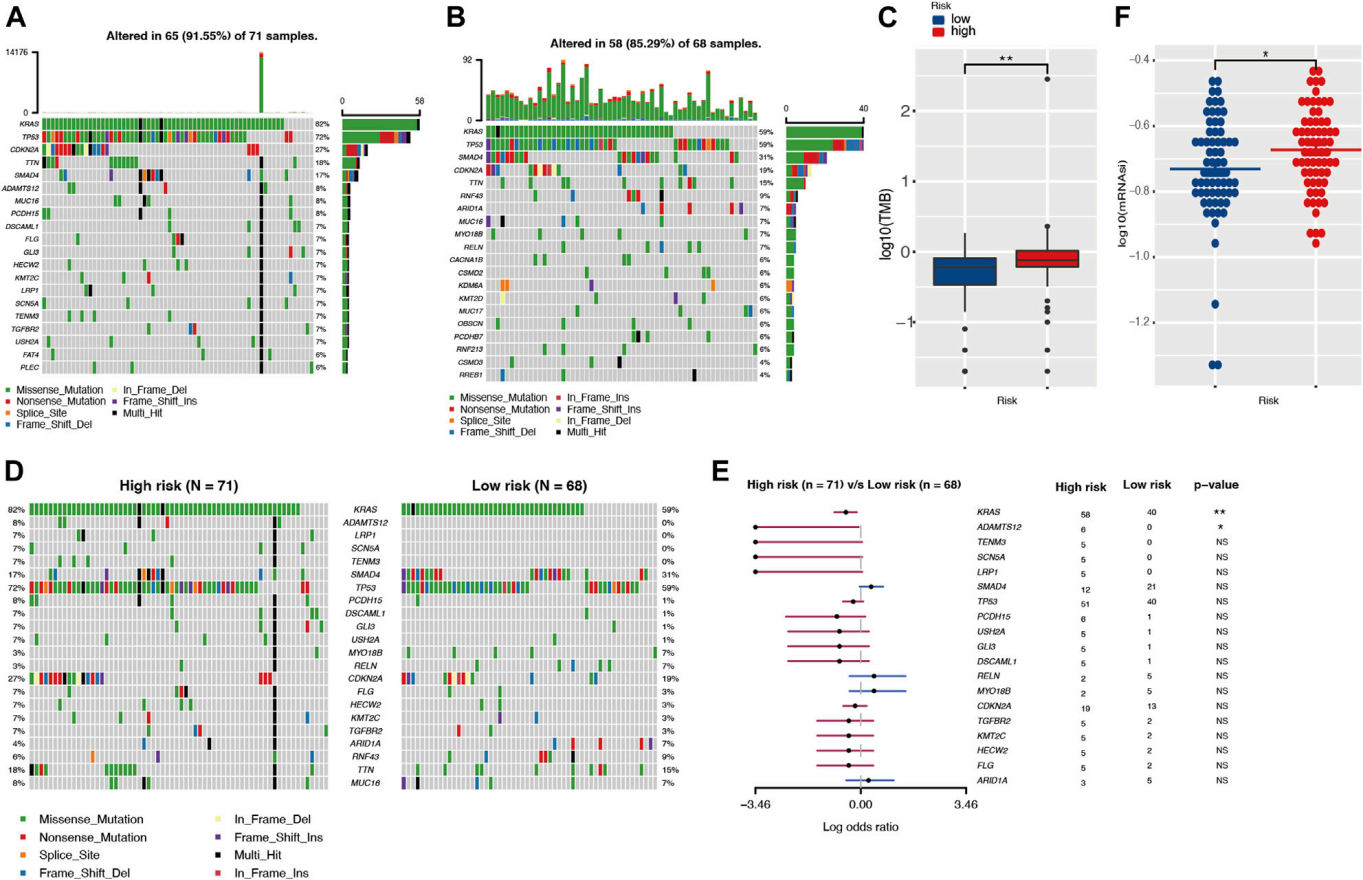
The landscape of somatic mutation and tumor immune microenvironment in high- and low-risk cohorts. **(A,B)** The waterfall plot shows the mutation distribution of the top 20 most frequently mutated genes in high- and low-risk groups, respectively. The central panel shows the types of mutations. The upper panel shows the mutation frequency in each PDAC sample. The bar plots on the left show the frequency and mutation type of genes. The bottom panel is the legend for the mutation types. **(C)** Comparison of TMBs between high- and low-risk groups. **(D,E)** The waterfall plot and forest plot display the differentially mutated genes between the two cohorts. **(F)** Comparison of stemness indices between high- and low-risk groups. TMB, tumor mutation burden.

The development of cancer is the result of somatic mutation and clonal selection. To explore the relationship between risk score and mutation status, we further analyzed and visualized somatic mutations between the high-risk and low-risk cohorts of TCGA. The results indicated that the top 20 genes with the highest mutation frequencies in both groups were similar and only differed in the ranking (Figures 7A,B). The genes with mutation rates above 10% included *KRAS, TP53, SMAD4, CDKN2A*, and *TNN*. Missense mutations were the most common type. In general, the high-risk cohort harbored more somatic mutations and showed a higher tumor mutation burden than the low-risk cohort (Figure 7C, *p* < 0.05). When comparing mutated genes in the two groups, only KRAS (82 vs. 59%, *p* < 0.01) and ADAMTS12 (8 vs. 0%, *p* < 0.05) were statistically different in mutation frequency (Figures 7D,E).

### Assessment of Tumor Immune Microenvironment and Stemness

Different tumor mutation burdens might suggest different immune cell infiltration statuses. To investigate whether patients in the high-risk group with higher TMB had more immune cell infiltration, we evaluated the immune microenvironment in both groups by CIBERSORT and TIMER algorithms (Newman et al., 2015; Li et al., 2020). The results suggested that there was no statistical difference in immune scores between the two groups (Supplementary Figure S7A). In parallel with this result, the TIMER assessment also showed that only CD4^+^ T cell infiltration might differ among the six immune cell types (Supplementary Figure S7B). The TIDE algorithm was exploited to predict responsiveness to immunotherapy and there was no statistical difference between the two groups (Supplementary Figure S7C), which further suggested that the two groups might have similar immune cell infiltration statuses. Tumor stemness was considered to be one of the key factors in tumor progression and was significantly associated with patient prognosis. In our investigation, we obtained the mRNA expression-based stemness index through one-class logistic regression (OCLR) machine learning algorithm. The high-risk group with a poorer prognosis had a higher stemness index (*p* < 0.05) (Figure 7F).

### Novel Drug Candidates Identified by CMap

**FIGURE 8:**
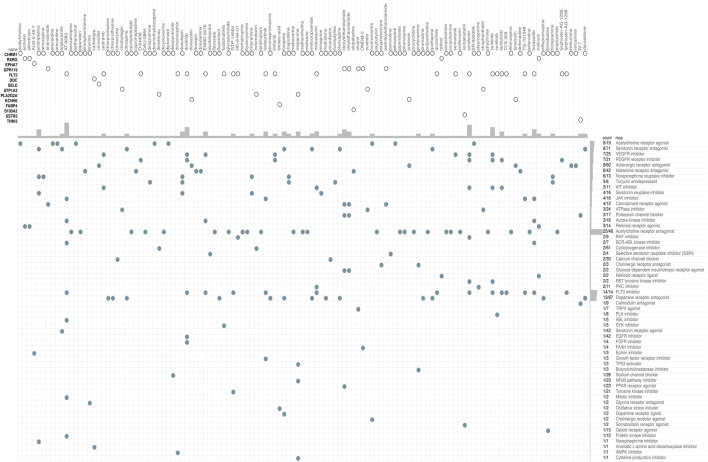
CMap database analysis identifies novel candidate drugs targeting the DEGs between high-risk and low-risk patients in the TCGA cohort. CMap, connectivity map.

The CMap database was used to identify potential drugs that might target DEGs between high risk and low risk patients in TCGA cohort (see Supplementary Table S5). As a result, 120 drug candidates were screened for 58 possible mechanisms of action (see Figure 8 and Supplementary Table S6). The top five drugs included AT-9283 (count = 7), sorafenib (count = 6), sunitinib (count = 6), dovitinib (count = 5), and tozasertib (count = 4). The top five possible mechanisms of action were Acetylcholine receptor antagonist (count = 25), FLT3 inhibitor (count = 14), Dopamine receptor antagonist (count = 13), Acetylcholine receptor agonist (count = 9) and Serotonin receptor antagonist (count = 8).

## Discussion

PDAC is a malignant gastrointestinal tumor with a 5 years survival rate of less than 10% (Mizrahi et al., 2020). One of the major obstacles to PDAC treatment is the high degree of heterogeneity. Although bulk sequencing has identified numerous genetic alterations in PDAC, it can only provide the average expression signal of the tissue tested, and its results may be confounded by tumor heterogeneity. Rapid progress in the development of scRNA-seq has allowed researchers to probe the heterogeneity at the single-cell level. Also, the resolution provided by scRNA-seq makes it possible to study the genomes of specific cell populations, which may allow researchers to uncover new and potentially unexpected biological discoveries (Grün and van Oudenaarden, 2015).

With the enormous development of genomics research in recent decades, a large amount of biological information has been accumulated, which has raised great expectations concerning its impact on personalized or precision medicine (Ginsburg and Phillips, 2018). In this study, we used scRNA-seq data to analyze the transcriptome differences between ductal cells in normal and PDAC tissues. A prognostic risk model constructed based on these DEGs effectively predicted the OS of PDAC patients. Moreover, it exhibited promising predictive efficacy even in different subgroups of patients, and this predictive power was further validated in external datasets. As an independent risk factor for PDAC, this risk score may serve as a useful complement to clinical features when clinicians assess patient survival.

Cancer is a genetic disease and the accumulation of somatic mutations is responsible for it (Martincorena, 2019). In our analysis, mutational profiles of PDAC were associated with survival. Patients in the high-risk group had more somatic mutations (i.e., higher TMB) (*p* < 0.05). The mutation frequencies of *KRAS* and *ADAMTS12* were significantly different between the high- and low-risk groups (*p* < 0.05). *KRAS* is one of the well-known driver genes of PDAC. Its somatic mutations are present in more than 90% of PDAC patients (Waters and Der, 2018). A growing number of studies have suggested that mutations in *KRAS* played an important role in tumor invasion, metastasis, and chemoresistance (Mueller et al., 2018; Buscail et al., 2020). In contrast, the role of *ADAMTS12* in PDAC was poorly understood, probably due to its relatively low mutation rate in PDAC. A recent study revealed that *ADAMTS12* was highly expressed in the PDAC stroma, which was closely associated with tumor progression (Robin et al., 2020). However, the effect of *ADAMTS12* on the tumor microenvironment needs to be further elucidated.

Tumors typically exhibit abnormalities in multiple cellular functions. Such dysfunctions were heterogeneous in the PDAC patients of our study. GSEA results indicated that the genetic profiles of several cellular functions in the high-risk group were significantly different from those in the low-risk group, including transcriptional regulation, proliferation, DNA damage repair, and metabolic patterns of cells. All of these functions are closely related to tumor development, and their heterogeneity may explain the survival differences between the two groups. Recently, a large number of studies have shown that tumor stemness is an essential mechanism of tumor resistance, recurrence, and metastasis, which makes it a significant reference in survival assessment (Batlle and Clevers, 2017; Miranda et al., 2019; Saygin et al., 2019). Poor patient survival has been linked to tumor stemness-associated traits (Valle et al., 2018). In this investigation, stemness scores were higher in the high-risk group, which may have led to worse survival outcomes.

In recent years, immunotherapy has made breakthroughs in the treatment of some solid tumors (Fukumura et al., 2018; Hegde and Chen, 2020). In particular, the anti-tumor efficacy of immune checkpoint inhibitors (ICI) has attracted a great deal of attention (Robert, 2020). However, the results of early trials using ICIs for PDAC were disappointing (Schizas et al., 2020). PDAC is known to be a typical “cold tumor”, lacking tumor-infiltrating immune cells and with most T cells in a depleted state (Bear et al., 2020). In our analysis, even though the high-risk group had a higher TMB, immune infiltration scores and responsiveness to immunotherapy did not differ significantly between the two groups. For PDAC with low immunogenicity, improving the effectiveness of immunotherapy remains promising but challenging. In addition to immunotherapy of PDAC, we identified seven targeted compounds associated with genes in the risk score model. They possess different mechanisms of action and have not been applied for PDAC treatment. These drug candidates may inspire ideas for future PDAC therapy.

In summary, by integrating scRNA-seq and bulk sequencing data, we established a risk score model for PDAC patients. The predictive score was an independent risk factor for PDAC, and it could be used for survival assessment. Future studies should focus on our predicted drug candidates and validate our findings.

## Data Availability

The original contributions presented in the study are included in the article/Supplementary Material, further inquiries can be directed to the corresponding authors.
