# Supplementary figures and images for: A Ductal-Cell-Related Risk Model Integrating Single-Cell and Bulk Sequencing Data Predicts the Prognosis of Patients With Pancreatic Adenocarcinoma
Source: Front Genet. 2022 Jan 3;12:763636. doi: 10.3389/fgene.2021.763636 (PMC8762279; doi:10.3389/fgene.2021.763636)

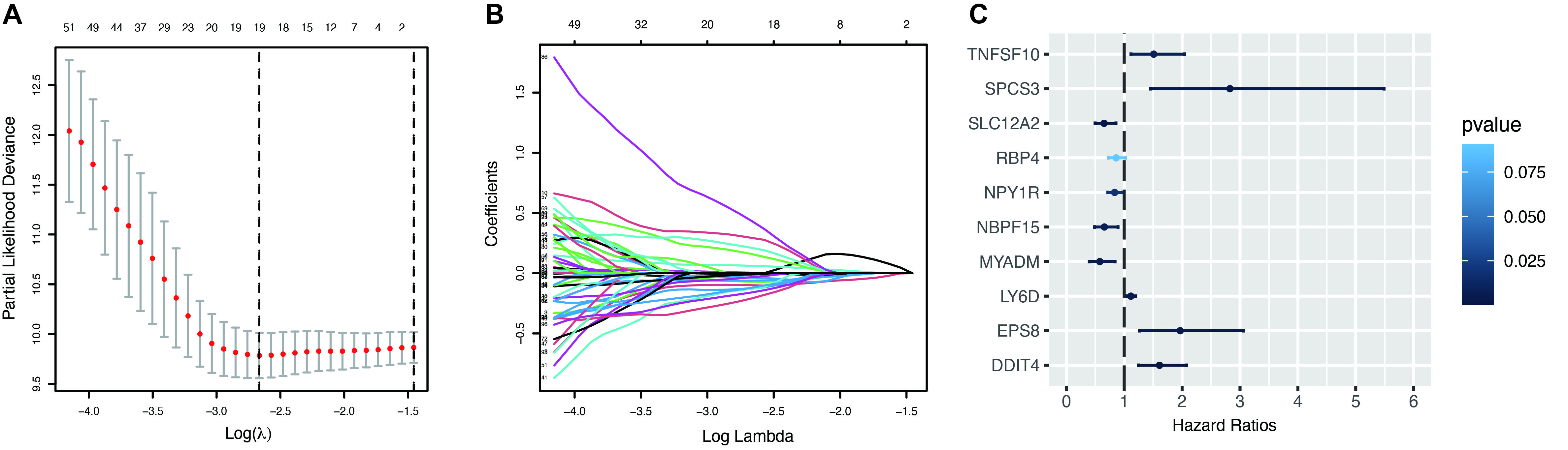

Supplement: Supplementary file 2 [file Image3.JPEG]

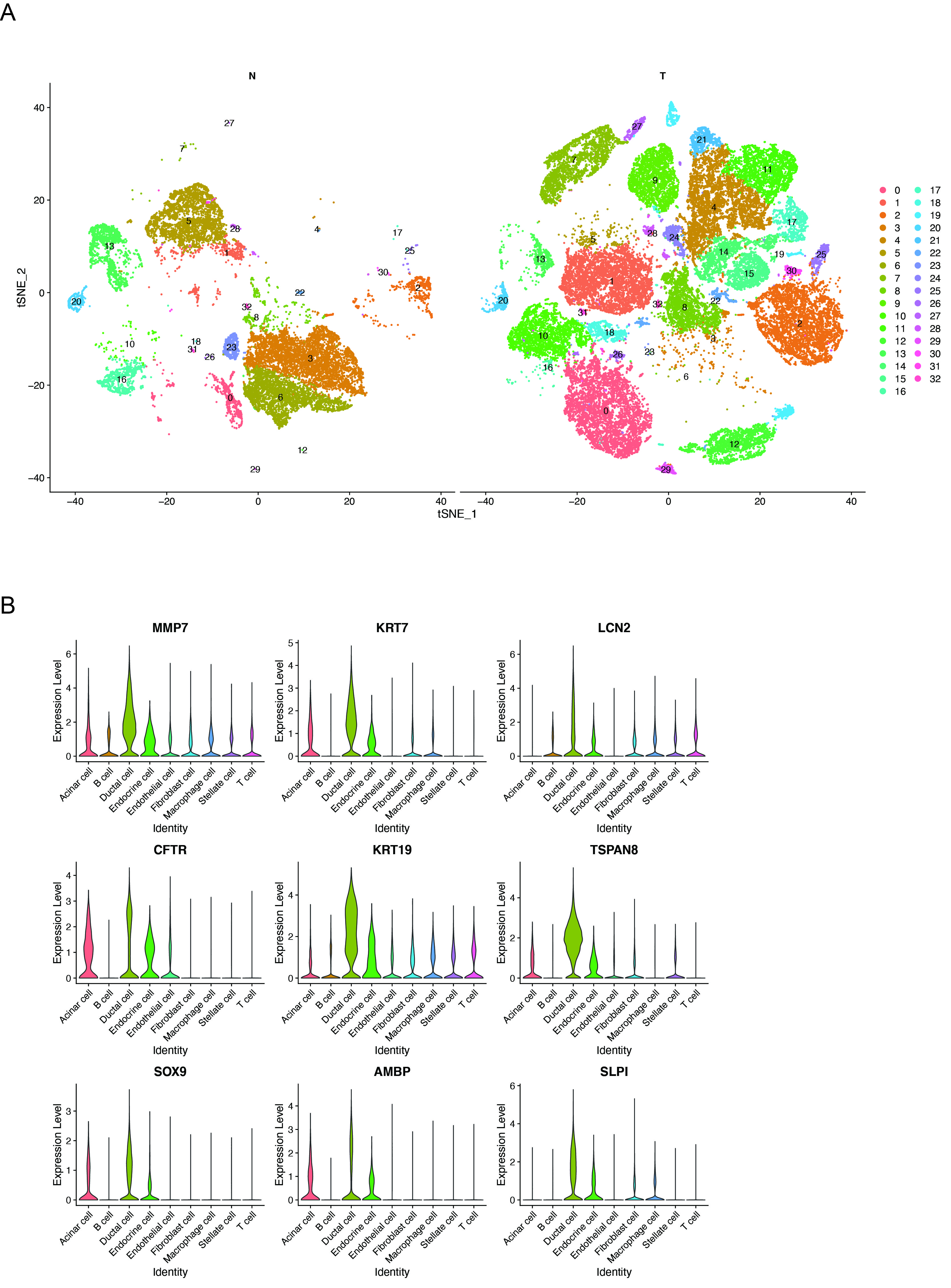

Supplement: Supplementary file 5 [file Image1.JPEG]

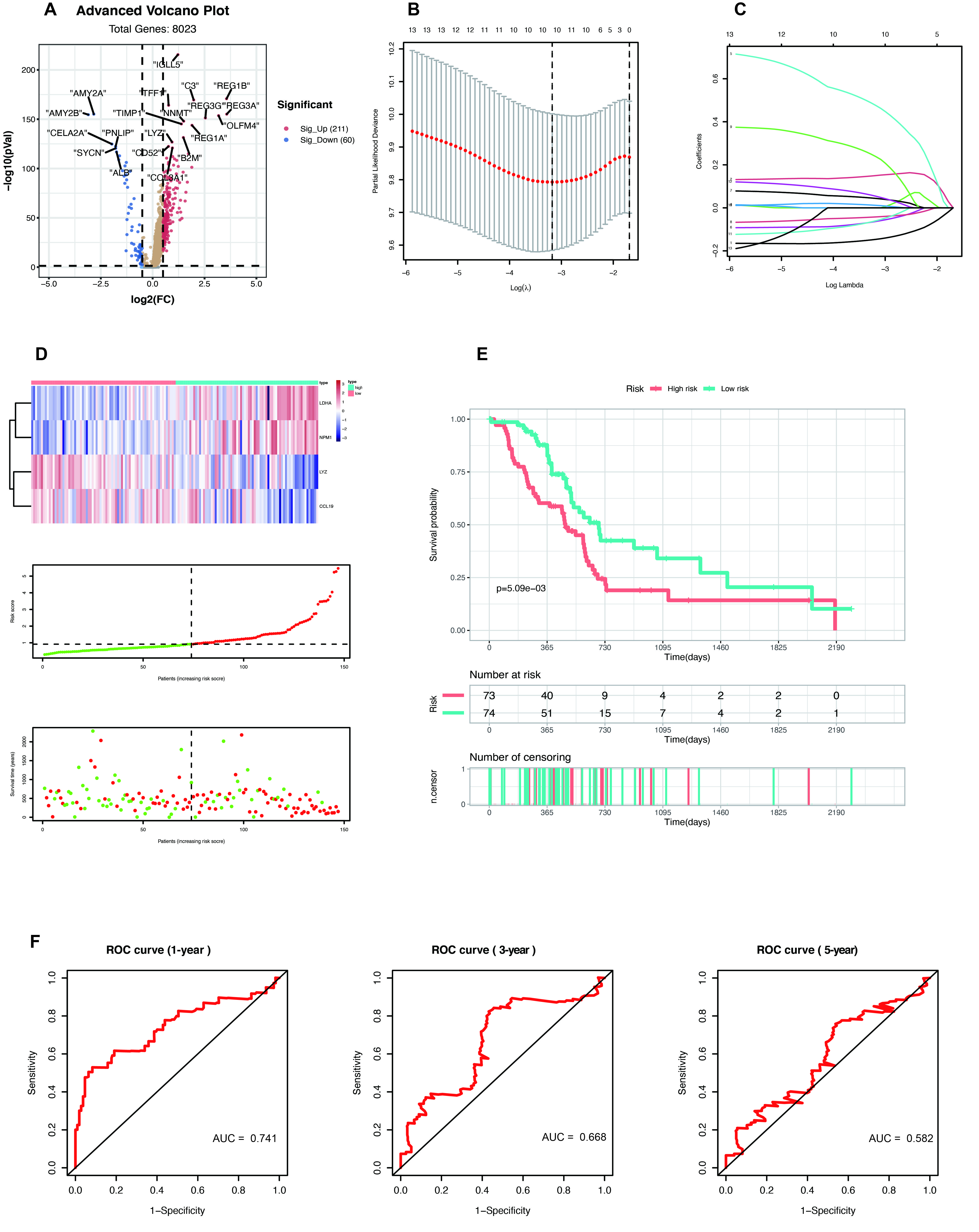

Supplement: Supplementary file 6 [file Image4.JPEG]

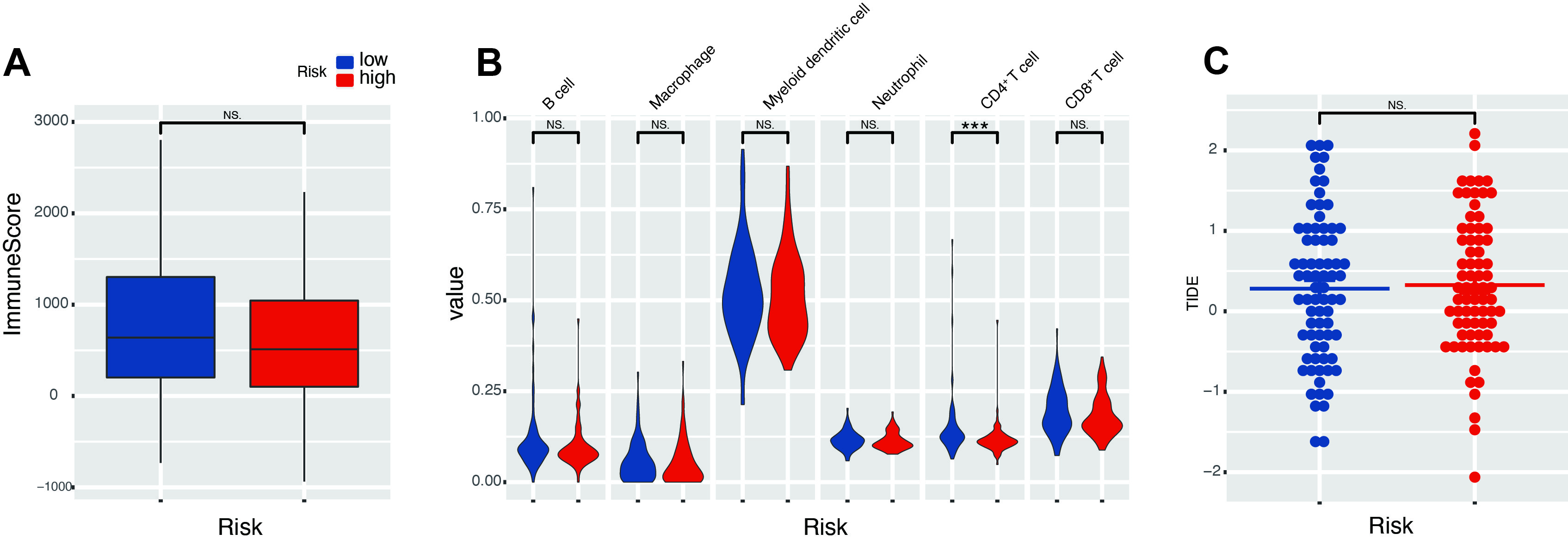

Supplement: Supplementary file 7 [file Image7.JPEG]

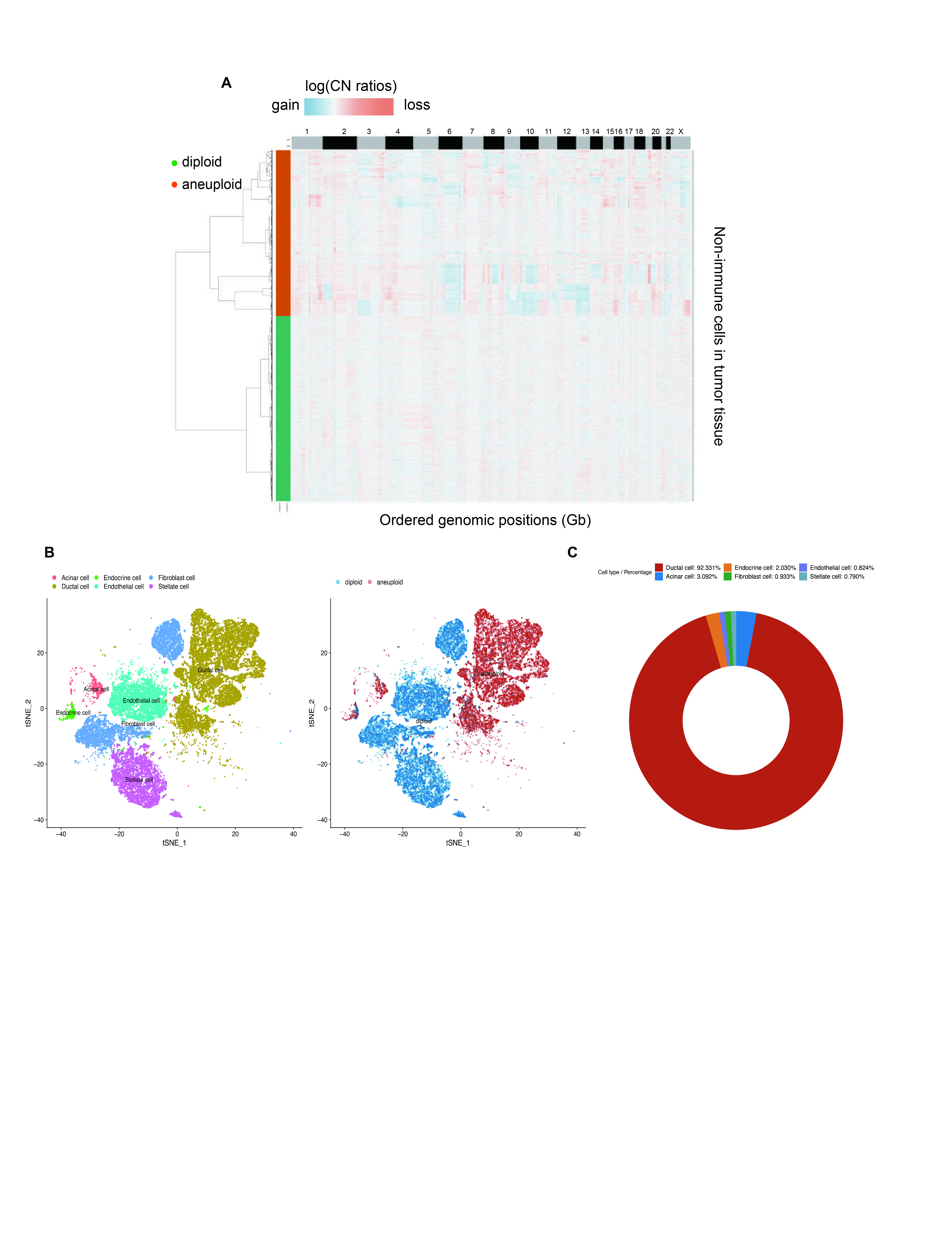

Supplement: Supplementary file 8 [file Image2.JPEG]

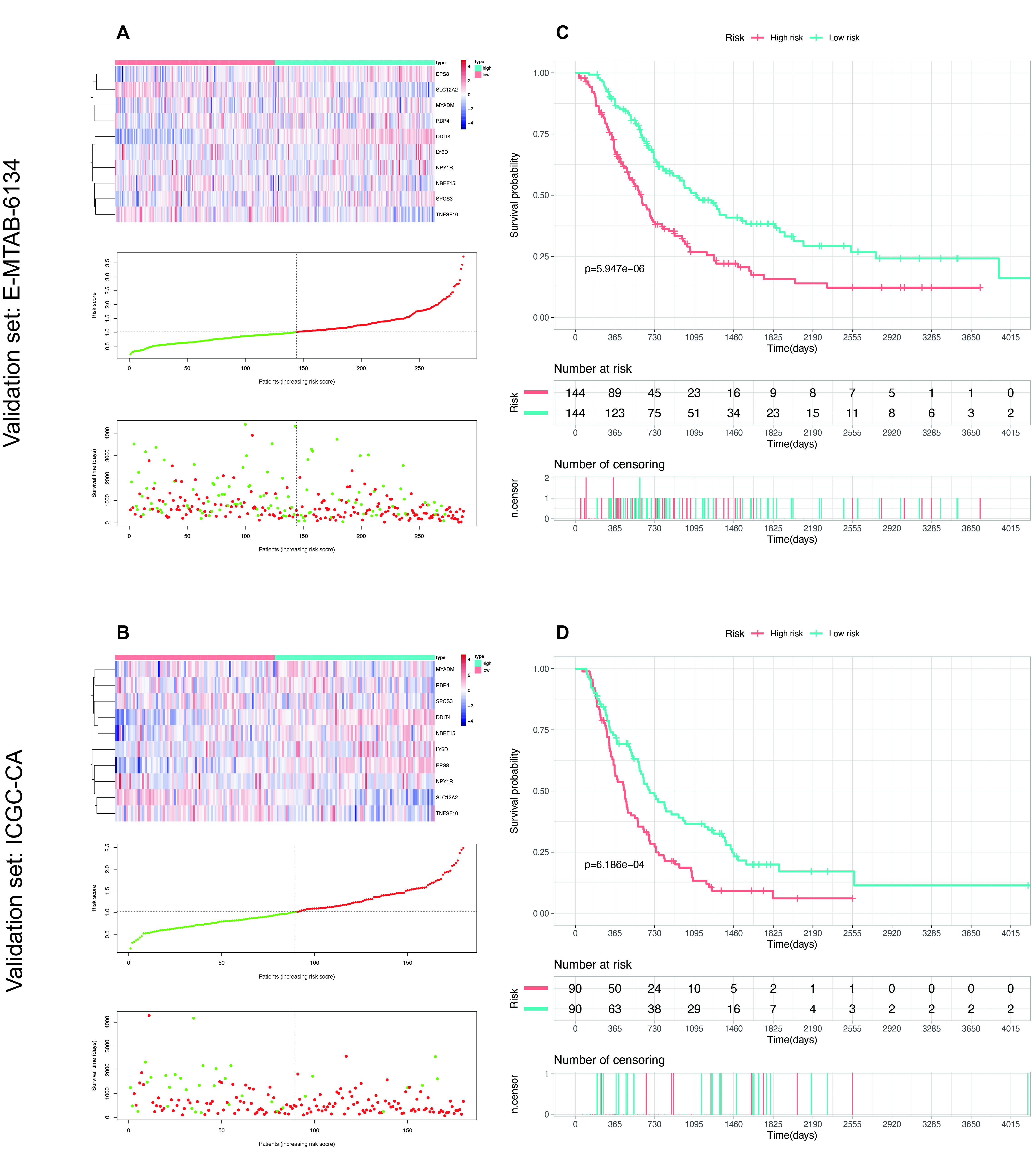

Supplement: Supplementary file 9 [file Image5.JPEG]

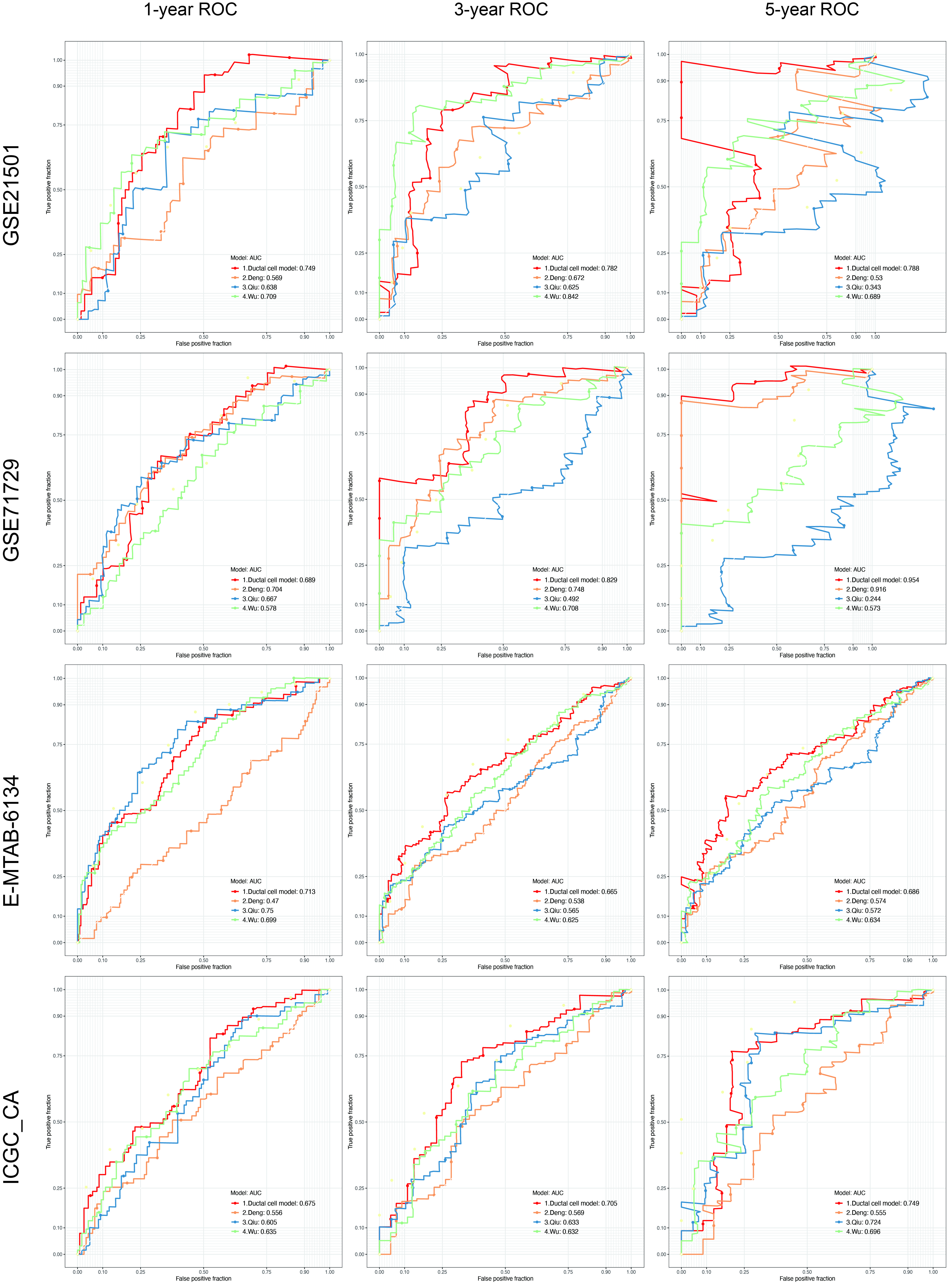

Supplement: Supplementary file 13 [file Image6.JPEG]
